# Evidence of a Causal Association Between Insulinemia and Endometrial Cancer: A Mendelian Randomization Analysis

**DOI:** 10.1093/jnci/djv178

**Published:** 2015-07-01

**Authors:** Kevin T. Nead, Stephen J. Sharp, Deborah J. Thompson, Jodie N. Painter, David B. Savage, Robert K. Semple, Adam Barker, The Australian National Endometrial Cancer Study Group (ANECS), John R. B. Perry, John Attia, Alison M. Dunning, Douglas F. Easton, Elizabeth Holliday, Luca A. Lotta, Tracy O’Mara, Mark McEvoy, Paul D. P. Pharoah, Rodney J. Scott, Amanda B. Spurdle, Claudia Langenberg, Nicholas J. Wareham, Robert A. Scott

**Affiliations:** **Affiliations of authors:**MRC Epidemiology Unit, University of Cambridge, Cambridge, UK (KTN, SJS, AB, JRBP, LAL, CL, NJW, RAS); Department of Radiation Oncology, University of Pennsylvania Perelman School of Medicine, Philadelphia, PA (KTN); Department of Public Health and Primary Care, University of Cambridge, Strangeways Research Laboratory, Cambridge, UK (DJT, DFE, PDPP); Queensland Institute of Medical Research, Brisbane, Australia (JNP, ANECS, TO, ABS); University of Cambridge Metabolic Research Laboratories, Wellcome Trust-Medical Research Council Institute of Metabolic Science, Cambridge, UK (DBS, RKS); Hunter Medical Research Institute, John Hunter Hospital, Newcastle, Australia (JA, EH, RJS); Centre for Clinical Epidemiology and Biostatistics, School of Medicine and Public Health, The University of Newcastle, Newcastle, Australia (JA, MM); Department of Oncology, University of Cambridge, Strangeways Research Laboratory, Cambridge, UK (AMD, DFE, PDPP); Centre for Information Based Medicine, School of Medicine and Public Health, University of Newcastle, Australia (EH, RJS).

## Abstract

**Background:**

Insulinemia and type 2 diabetes (T2D) have been associated with endometrial cancer risk in numerous observational studies. However, the causality of these associations is uncertain. Here we use a Mendelian randomization (MR) approach to assess whether insulinemia and T2D are causally associated with endometrial cancer.

**Methods:**

We used single nucleotide polymorphisms (SNPs) associated with T2D (49 variants), fasting glucose (36 variants), fasting insulin (18 variants), early insulin secretion (17 variants), and body mass index (BMI) (32 variants) as instrumental variables in MR analyses. We calculated MR estimates for each risk factor with endometrial cancer using an inverse-variance weighted method with SNP-endometrial cancer associations from 1287 case patients and 8273 control participants.

**Results:**

Genetically predicted higher fasting insulin levels were associated with greater risk of endometrial cancer (odds ratio [OR] per standard deviation = 2.34, 95% confidence internal [CI] = 1.06 to 5.14, *P* = .03). Consistently, genetically predicted higher 30-minute postchallenge insulin levels were also associated with endometrial cancer risk (OR = 1.40, 95% CI = 1.12 to 1.76, *P* = .003). We observed no associations between genetic risk of type 2 diabetes (OR = 0.91, 95% CI = 0.79 to 1.04, *P* = .16) or higher fasting glucose (OR = 1.00, 95% CI = 0.67 to 1.50, *P* = .99) and endometrial cancer. In contrast, endometrial cancer risk was higher in individuals with genetically predicted higher BMI (OR = 3.86, 95% CI = 2.24 to 6.64, *P* = 1.2x10^-6^).

**Conclusion:**

This study provides evidence to support a causal association of higher insulin levels, independently of BMI, with endometrial cancer risk.

Metabolic diseases such as type 2 diabetes (T2D) are an established and growing worldwide concern with important public health implications (1,2). In particular, insulin concentrations and T2D have been associated with higher incidence and mortality from numerous cancers in observational studies (3,4). Insulin resistance and elevated C-peptide, a marker of insulin release, confer a higher risk of endometrial cancer (5,6). Furthermore, a meta-analysis of 16 studies showed that women with diabetes had a greater-than-two-fold higher endometrial cancer risk (7). However, insulin resistance, T2D, and endometrial cancer share common risk factors, such as higher body mass index (BMI), which may confound observational epidemiological studies (8). Therefore, it is uncertain if the associations between hyperinsulinemia and T2D with endometrial cancer are causal.

Where confounding is suspected in epidemiological studies and a randomized controlled trial is difficult to implement, Mendelian randomization provides a promising alternative (9). Using the example of fasting insulin, this method is akin to a “genetically randomized controlled trial” where, under Mendel’s law of independent assortment, individuals are randomly assigned to varying levels of insulin, independently of confounding variables. This approach is dependent on three assumptions of genetic variants used as instrumental variables: first, that the genetic variants are associated with the risk factor (ie, insulin); secondly, that the variants are not associated with other confounders; and thirdly, that the variants are independent of the outcome given the risk factor and confounders (9). Using this design, a genetically conferred elevation in insulinemia should result in a higher risk of endometrial cancer only if insulinemia is truly on the causal pathway for this disease.

Recently, a number of variants have been identified to be associated with fasting and postchallenge insulin levels (10). Here we implement a Mendelian randomization approach to test whether hyperinsulinemia, T2D, and related traits are causally related to endometrial cancer. Using data from genome-wide association studies (GWAS) of endometrial cancer, we examine the association of single nucleotide polymorphisms (SNPs) for fasting insulin (FI), postchallenge insulin, fasting glucose (FG), BMI, and T2D with endometrial cancer.

## Methods

### Instrumental Variable Definition

We utilized genetic variants associated with each risk factor (FI, FG, postchallenge insulin, T2D, and BMI). Where available, we included all variants associated with FI (11), T2D (12–15), FG (11), and BMI (16) at genome-wide statistical significance thresholds (*P* < 5 x 10^–8^) in previously published large-scale genome-wide meta-analyses in individuals of European ancestry. For postchallenge insulin secretion, where discovery sample sizes are smaller (17), variants from a genetic score shown to be strongly associated with insulin secretion (10) were included. In recently published large-scale meta-analyses (17), two (of 19) variants previously included in the insulin secretion score (near *DGKB* and *TFB1M*) had an opposite direction of effect to that expected (albeit with statistically nonsignificant associations) and, as such, were excluded from analyses as detailed in Supplementary Table 1 (available online). As the effects of *FTO* variants on FI and T2D appear entirely mediated through higher BMI (11,18), variants from this locus were excluded from the FI- and T2D-associated SNPs to minimize potential confounding by BMI. In total, 18 FI-associated variants, 49 T2D-associated variants, 36 FG-associated variants, 32 BMI-associated variants, and 17 variants associated with early postchallenge insulin secretion (10) were included in analyses (Supplementary Table 1, available online).

### Summary Statistic Mendelian Randomization

For each risk factor we estimated “SNP-risk factor” and “SNP-endometrial cancer” associations (and their standard errors) to calculate individual SNP estimates of each “risk factor–endometrial cancer” association, which were then combined using an inverse-variance weighted approach (19). Using this method, the association of each genetically predicted risk factor with endometrial cancer was estimated as a mean of individual SNP effects on endometrial cancer with each variant weighted by its effect on the relevant exposure (ie, FI). As one of the SNPs associated with postchallenge insulin secretion (rs7903146) was also associated with fasting insulin, we performed a sensitivity analysis excluding this variant from the postchallenge insulin secretion-associated SNPs. The effect sizes for the association of genetic variants and their respective traits were taken from replication-only effect size estimates from previously published large GWAS for FI, FG, BMI, and the presence of T2D (11,15,16) to obtain the most accurate estimates available. As variants associated with insulin secretion were identified through a range of sources (10), they were weighted according to the magnitude of their association (in discovery analyses) with 30-minute insulin in recent meta-analyses (17). All weighting factors are shown in Supplementary Table 1 (available online).

We examined the potential for confounding by investigating the association of unweighted genetic scores comprising the above variants with a range of potential confounders in the Fenland study (20) and with 30-minute insulin in the Ely study (21). These included age, age at menarche and menopause, BMI, FG, FI, 30-minute insulin, as well as with self-reported physical activity levels (22) and total energy intake, by linear regression. We also investigated the association of genetic scores with level of education (whether the highest level of education was up to a General Certificate of Secondary Education [GCSE] or whether A-level or degree level qualifications were obtained) as a proxy for socioeconomic status, by logistic regression. We additionally tested the association of the genetic scores with smoking status (never vs ever).

### Endometrial Cancer Associations

The associations of the selected variants with endometrial cancer were taken from an updated GWAS of endometrial cancer (23). All case patients were of endometroid histology and were derived from the Australian National Endometrial Cancer Study (ANECS; n = 606 case patients) or the Studies of Epidemiology and Risk factors in Cancer Heredity study (SEARCH; n = 681 case patients) (23). UK control participants (n = 5190) were derived from the Wellcome Trust Case Control Consortium 2 (WTCCC2) (24), and Australian control participants were from the parents of the twins in the Brisbane Adolescent Twin Study (n = 1846) (25) and from the Hunter Community Study (n = 1237) (26). All individuals provided written informed consent, and respective institutional review board approval was granted. Further details on each study are provided in the Supplementary Material (available online). In total, the endometrial cancer study comprised 1287 case patients and 8273 control participants. Studies from the UK and Australia were analyzed separately and combined using a fixed-effect inverse-variance weighted meta-analysis, with the Australian analysis adjusted for the first two principal components and the UK analysis adjusted for the first three principal components to account for latent population stratification.

Genotyping for the endometrial cancer case patients was carried out using the Human 610K array on the Illumina Infinium platform. Variants not meeting the following quality control thresholds were excluded: 1) call rate of 95% or greater with minor allele frequency of 0.05 or greater or call rate of 99% or greater with minor allele frequency under 0.05; 2) Hardy-Weinberg equilibrium *P* value of greater than 10^–12^ for case patients or *P* value of greater than 10^–7^ for control participants. Genotype imputation using HapMap 2 CEU data (27) as the reference panel was performed using MACH software (28). Imputed SNPs with an imputation R^2^ of less than 0.6 were excluded.

### Scaling of Results for Each Genetically Predicted Risk Factor

For continuous variables, the results of the weighted method are scaled per standard deviation (SD) of log-FI, FG, BMI, and per SD of log 30-minute insulin. Standard deviations were derived from the population-based Fenland or Ely studies (20,21) and shown in Table 1. Therefore, for continuous exposures, the effect sizes represent the odds ratio of endometrial cancer per genetically predicted SD increase in the exposure. When T2D is the exposure, the effect size represents the odds ratio of endometrial cancer per genetically predicted increase of one in the log-odds of T2D.

**Table 1.** Associations with endometrial cancer per genetically predicted SD increase of each risk factor

Risk factor	SD	OR per genetically predicted SD (95% CI)	P*
Body mass index, kg/m^2^	4.81	3.86 (2.24 to 6.64)	1.2x10^-6^
Fasting glucose, mmol/L	0.65	1.00 (0.67 to 1.50)	.99
Fasting insulin, ln(pmol/L)	0.60	2.34 (1.06 to 5.14)	.03
Early insulin secretion, ln(pmol/L)	0.58	1.40 (1.12 to 1.76)	.003
Type 2 diabetes, log-odds	1	0.91 (0.79 to 1.04)	.16

* Inverse-variance weighted model. All statistical tests were two-sided. CI = confidence interval; OR = odds ratio.

Individual variants were examined for association with endometrial cancer using Bonferroni-corrected two-sided *P* values (eg, for T2D, α = 0.05/49). Tests were otherwise considered statistically significant if the two-sided *P* value was under .05. All analyses were performed using Stata version 12.0 (StataCorp, College Station, TX) or as otherwise indicated.

## Results

We observed an association of variants associated with higher fasting insulin with higher risk of endometrial cancer (odds ratio [OR] per genetically predicted SD of log-FI = 2.34, 95% confidence interval [CI] = 1.06 to 5.14, *P* = .03) (Table 1). We found a directionally consistent association of genetically predicted higher postchallenge insulin levels with a higher risk of endometrial cancer (OR = 1.40, 95% CI = 1.12 to 1.76, *P* = .003). After exclusion of the rs7903146 variant, which was also associated with FI, from the postchallenge insulin-associated secretion SNPs the result remained statistically significant (OR = 1.32, 95% CI = 1.05 to 1.67, *P* = .02). No association was found for T2D (OR = 0.91, 95% CI = 0.79 to 1.04, *P* = .16) or FG (OR = 1.00, 95% CI = 0.67 to 1.50, *P* = .99) with endometrial cancer (Table 1). We found a more-than-three-fold higher endometrial cancer risk per genetically predicted SD increase in BMI (OR = 3.86, 95% CI = 2.24 to 6.64, *P* = 1.2x10^-6^) (Table 1).

For the fasting insulin, postchallenge insulin, and BMI-associated variants, we evaluated the potential for individual pleiotropic outlier SNPs to underlie associations, but we observed no clear outliers (Supplementary Figure 1, A-C, available online). Three individual variants reached Bonferroni-corrected statistical significance thresholds for association with endometrial cancer (Supplementary Table 1, available online). The BMI-increasing allele at *FTO* was associated with higher risk of endometrial cancer (OR = 1.16, 95% CI = 1.07 to 1.27, *P* = 5.6×10^–4^), which may reflect the larger effect size of *FTO* variants on BMI than other variants (Supplementary Figure 1C, available online). Genetically predicted BMI remained associated with higher risk of endometrial cancer after excluding the *FTO* variant (OR = 3.19, 95% CI = 1.70 to 6.03, *P* = 3.2×10^–4^). The T2D risk-increasing allele of *HNF1B* variant rs11651052 (A) was protective against endometrial cancer (OR = 0.82, 95% CI = 0.76 to 0.89, *P* = 3.7×10^–6^), while the FG-raising G-allele of *ADRA2A* variant rs10885122 (OR = 1.32, 95% CI = 1.13 to 1.54, *P* = 3.6×10^–4^) was associated with a higher endometrial cancer risk. Removing these variants did not change the associations of genetically predicted fasting glucose or T2D risk with endometrial cancer (Table 2).

**Table 2.** Associations with endometrial cancer per genetically predicted SD of each risk factor from sensitivity analyses, excluding single nucleotide polymorphisms exceeding Bonferroni-corrected thresholds for association with endometrial cancer

Risk factor	SD	OR per genetically predicted SD (95% CI)	P*
Body mass index, kg/m^2^	4.81	3.20 (1.70 to 6.03)	<.001
Fasting glucose, mmol/L	0.65	0.94 (0.62 to 1.41)	.76
Type 2 diabetes, log-odds	1	0.97 (0.85 to 1.12)	.71

* Inverse-variance weighted model. All statistical tests were two-sided. CI = confidence interval; OR = odds ratio.

**Figure 1.**
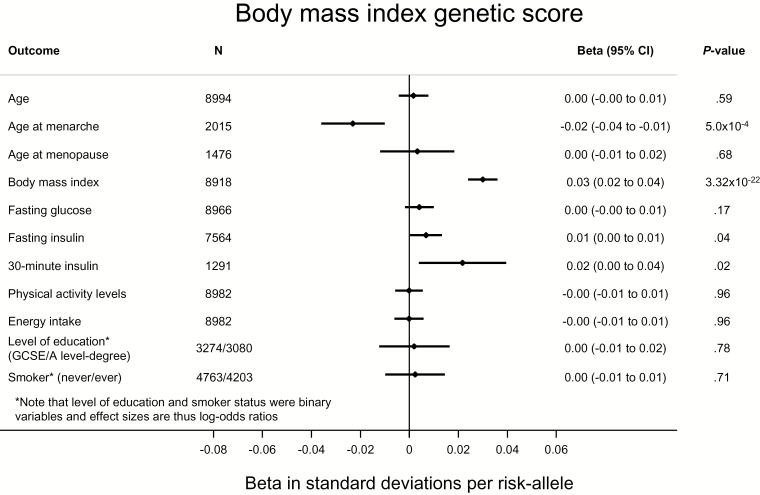
Association of a genetic score of body mass index (BMI)–associated single nucleotide polymorphisms with BMI and a range of potential confounders in the Fenland study (20). Thirty-minute insulin was only available in the Ely study (21), so the sample size is smaller. Given 55 tests (α = 9.1x10^-4^), the score was associated with BMI and with age at menarche (29). Associations with quantitative traits were tested by linear regression and with binary traits by logistic regression. All statistical tests were two-sided.

**Figure 2.**
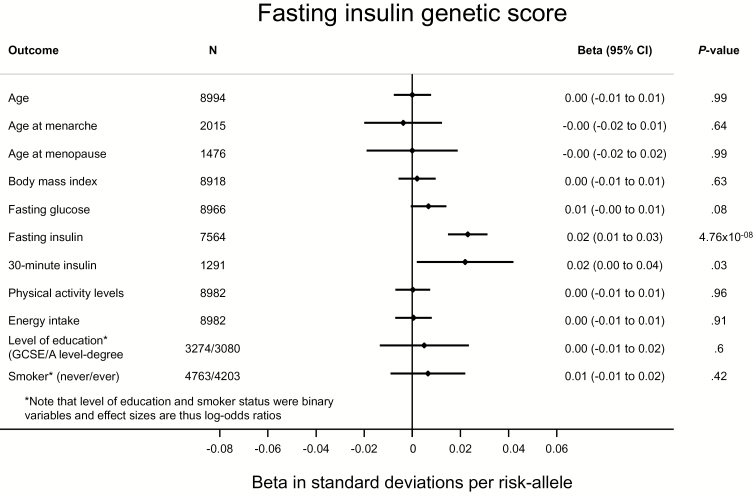
Association of a genetic score of fasting insulin–associated single nucleotide polymorphisms with fasting insulin and a range of potential confounders in the Fenland study (20). Thirty-minute insulin was only available in the Ely study (21), so the sample size is smaller. Given 55 tests (α = 9.1x10^-4^), the score was only associated with fasting insulin. Associations with quantitative traits were tested by linear regression and with binary traits by logistic regression. All statistical tests were two-sided.

**Figure 3.**
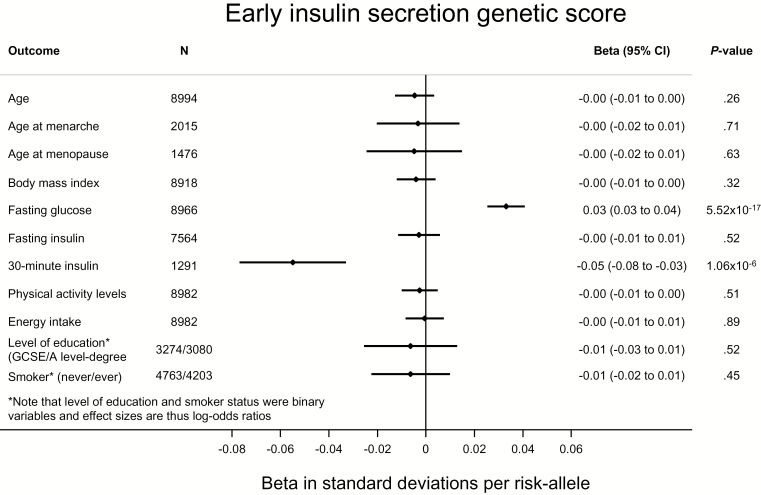
Association of a genetic score of insulin secretion–associated single nucleotide polymorphisms with early insulin secretion and a range of potential confounders in the Fenland study (20). Thirty-minute insulin was only available in the Ely study (21), so the sample size is smaller. Given 55 tests (α = 9.1x10^-4^), the score was only associated with early insulin secretion and with fasting glucose. Associations with quantitative traits were tested by linear regression and with binary traits by logistic regression. All statistical tests were two-sided.

To further investigate the potential for pleiotropy, and the extent to which our Mendelian randomization was “randomized,” we investigated the association of genetic scores comprising risk factor–associated SNPs with a range of potential confounders. We found no compelling evidence for the associations we report being driven by these confounders (Figures 1–3; Supplementary Figures 2 and 3, available online). In most instances, genetic scores were associated with their relevant risk factors, but not with other traits. We did observe an association of the BMI score with age at menarche (Figure 1) (29) and of the insulin secretion score with fasting glucose (Figure 3) (10). However, importantly, neither the fasting insulin (*P* = .63) nor the insulin secretion score (*P* = .32) were associated with BMI (Figures 2 and 3).

## Discussion

The present study provides evidence for a causal association between insulinemia and endometrial cancer that is independent of known confounding factors, including BMI. This is supported by two nonoverlapping sets of genetic variants associated with fasting and postchallenge insulin. Interestingly, we did not observe a causal association for T2D, nor with fasting glucose, despite the strong and consistent association of T2D with endometrial cancer demonstrated in observational studies.

A limited number of epidemiological studies have directly examined the association between hyperinsulinemia and endometrial cancer. Independently of BMI, a greater-than-two-fold higher risk of endometrial cancer has been demonstrated when comparing women in the highest quartile of FI to those in the lowest quartile (5), and a more-than-four-fold higher risk of endometrial cancer has been observed among women in the highest compared with lowest quintile of C-peptide levels (6). An additional study examined insulin resistance using the homeostasis model assessment ratio (HOMA-IR) and found that, compared with the lowest quartile, women in the highest quartile had a two-fold higher risk of endometrial cancer even after adjustment for waist-hip ratio (30). Finally, higher FI has been associated with increased endometrial cancer stage and faster disease progression (31).

Our findings that a genetically predicted increase in FI raises endometrial cancer risk, while a genetically predicted increase in postchallenge insulin levels (not associated with insulin resistance (10) or with FI [Figure 3]) also raises endometrial cancer risk, jointly support a causal role for hyperinsulinemia in the etiology of endometrial cancer. Hyperinsulinemia is a leading hypothesis for the epidemiological association of T2D and endometrial cancer (7), with multiple plausible mechanisms to explain this observation. First, insulin decreases levels of sex hormone binding globulin (SHBG) by inhibiting its production in the liver (32). As SHBG typically binds estrogens and other sex hormones, lower levels of SHBG result in an elevation of bioavailable estrogens (33,34). Consequently, postmenopausal diabetic women have been observed to have higher levels of urinary estrogens as compared with postmenopausal nondiabetic women, independent of body weight (35). As with the peripheral conversion of androgens to free estrogens when excess adipose tissue is present, elevated estrogens secondary to hyperinsulinemia increase endometrial cell proliferation and decrease apoptosis, resulting in an increased endometrial cancer risk (36). Accordingly, there is some evidence that genetic variants that have been shown to increase SHBG levels are also associated with a lower risk of endometrial cancer (37). Secondly, hyperinsulinemia leads to decreased levels of insulin like growth factor binding protein, which results in elevated levels of free insulin-like growth factor 1 (IGF-1) (7,38). IGF-1 receptors are present in endometrial tissue and have been shown to stimulate endometrial cell proliferation (39,40). Insulin itself may further contribute to endometrial cell proliferation, as it has been shown to act as an analogue of IGF-1 in endometrial tissue (41). These direct actions of insulin may also contribute to the association of type 1 diabetes and endometrial cancer (7), as mediated through the effects of exogenous insulin administration (42). Recent reports on individuals carrying rare loss-of-function *PTEN* variants with a predisposition to a range of cancers (including endometrial) despite lower insulin levels may appear in conflict with our results (43). However, *PTEN* is a negative regulator of insulin signaling, such that PTEN deficiency results in increased insulin signaling (44).

The association between higher BMI and endometrial cancer is well established in epidemiological studies (45). Excess adipose tissue results in greater peripheral aromatization of circulating androgens to bioavailable estrogen. Elevated estrogen levels have been shown to increase endometrial cancer risk, as estrogens are capable of stimulating endometrial cell proliferation and inhibiting apoptosis (36). Additionally, even in the absence of elevations in circulating estrogens, obesity is a risk factor for endometrial cancer through obesity-induced insulin resistance and the resultant higher insulin levels (46), as discussed above. It has been demonstrated that individual BMI-increasing variants, particularly in *FTO*, are associated with higher endometrial cancer risk (47,48). Here we extend these findings by using multiple BMI-associated variants, augmenting the evidence that the association between BMI and endometrial cancer is causal. In the current analysis, a genetically predicted SD increase in BMI (4.81kg/m^2^) was associated with a 3.86-fold higher risk of endometrial cancer. This finding is consistent with estimates from a meta-analysis of conventional epidemiological studies (45) where a 5kg/m^2^ increase in BMI was associated with a three-fold higher risk of endometrial cancer among individuals with a BMI above 28kg/m^2^. As the risk of endometrial cancer has been shown to be positively associated with the mean BMI in a population (49), a causal association between BMI and endometrial cancer indicates a likely increased future burden of disease among progressively obese populations.

The current study did not demonstrate an association between the T2D variants and endometrial cancer. One possibility is that we were underpowered to detect a true statistically significant association. However, this study was adequately powered to detect an association with genetically predicted elevations in FI, and the FI SNPs explained only around 1% of the variance in FI (11), while the T2D variants explained approximately 5% of the variance in T2D (15). Furthermore, the T2D point estimate in this study was less than one, whereas a meta-analysis of epidemiological studies predicts a two-fold increased risk of endometrial cancer (7). While recent analyses highlight that loci associated with T2D have a diverse range of underlying mechanisms (50), the point estimate for the T2D variants may reflect that many of these SNPs are associated with lower insulin secretion. Thus, our results suggest that the association of T2D with the risk of endometrial cancer is driven by the hyperinsulinemia observed in T2D, rather than hyperglycemia per se.

A primary assumption of this analysis is that the selected genetic variants are indeed associated with the exposure being tested (9). Therefore, we only used variants associated with the relevant exposure at genome-wide significance from hypothesis-free genome-wide meta-analyses. While this level of association was not available for variants associated with insulin secretion, we have previously demonstrated that this genetic score was strongly, and specifically, associated with higher postchallenge insulin levels (10).

A second assumption of this analysis is that variants are associated with endometrial cancer only through the exposure and are unconfounded by pleiotropy (9). We saw no convincing evidence of confounding in analyses investigating pleiotropy (Figures 1–3; Supplementary Figures 2 and 3, available online). While not possible to exclude confounding by unknown confounders, the use of multiple independent variants acting through different pathways decreases the likelihood of confounded instrumental variable associations (9,51). Importantly, we saw no association of either the fasting or postchallenge insulin scores with BMI (Figures 2 and 3).

As with any Mendelian randomization analysis, there are potential limitations to our findings, including the limited trait variance explained by genetic variants, thus restricting statistical power. This is particularly relevant for null findings, where wide confidence intervals leave uncertainty over the presence of a small causal effect. Thus, further genetic discovery efforts combined with larger studies of disease outcomes will further improve the utility and precision of Mendelian randomization analyses.

In conclusion, this study provides evidence for a causal role of higher insulin levels in the etiology of endometrial cancer. These findings are consistent with proposed mechanisms for the association of the T2D phenotype with endometrial cancer. Given the epidemics of obesity and insulin resistance, these findings indicate a growing importance of hyperinsulinemia on endometrial cancer incidence. The potential contribution of exogenous insulin to endometrial cancer development remains unknown but warrants consideration. Future studies should examine whether hyperinsulinemia and exogenous insulin have a causal role in disease progression and whether better insulin control results in decreased incidence or improved outcomes in endometrial cancer.

## Funding

This study was supported by Medical Research Council (MRC) grant MC_UU_12015/1 and by the Innovative Medicines Initiative Joint Undertaking under European Medical Information Framework (EMIF) grant agreement No. 115372 (contributions from the European Union’s Seventh Framework Programme [FP7/2007–2013] and EFPIA companies).

Australian National Endometrial Cancer Study (ANECS) recruitment was supported by project grants from the National Health and Medical Research Council of Australia (ID#339435), The Cancer Council Queensland (ID#4196615), and Cancer Council Tasmania (ID#403031 and ID#457636). Studies of Epidemiology and Risk factors in Cancer Heredity study (SEARCH) recruitment was funded by a programme grant from Cancer Research UK (C490/A10124). Case genotyping was supported by the National Health and Medical Research Council (ID#552402). Control data was generated by the Wellcome Trust Case Control Consortium (WTCCC), and a full list of the investigators who contributed to the generation of the data is available from the WTCCC website. We acknowledge use of DNA from the British 1958 Birth Cohort collection, funded by the Medical Research Council grant G0000934 and the Wellcome Trust grant 068545/Z/02. Funding for this project was provided by the Wellcome Trust under award 085475. Recruitment of the QIMR control participants was supported by the National Health and Medical Research Council of Australia (NHMRC). The University of Newcastle, the Gladys M. Brawn Senior Research Fellowship scheme, The Vincent Fairfax Family Foundation, the Hunter Medical Research Institute, and the Hunter Area Pathology Service all contributed towards the costs of establishing the Hunter Community Study.

KTN was supported by the Gates Cambridge Trust. RKS is supported by the Wellcome Trust (grant number WT098498). ABS is supported by the National Health and Medical Research Council (NHMRC) Fellowship Scheme. DFE is a Principal Research Fellow of Cancer Research UK. AMD is supported by the Joseph Mitchell Trust.

## Supplementary Material

Supplementary Data

## Notes

The study funders had no role in the design of the study; the collection, analysis, or interpretation of the data; the writing of the manuscript; nor the decision to submit the manuscript for publication.

NECS, SEARCH, QIMR, and the HCS thank the many individuals who participated in the research studies, Penny Webb, Kaltin Ferguson, Felicity Lose, Shahana Ahmed, Catherine Healey, Kyriaki Michailidou and the ANECS research team, the Eastern Cancer Registration and Information Centre and the SEARCH research team, and the numerous institutions and their staff who supported recruitment. NECS, SEARCH, QIMR, and the HCS acknowledge the contribution of our clinical and scientific collaborators and their staff. See http://www.anecs.org.au/ for full listing of the ANECS Group and other contributors to ANECS. SEARCH collaborators include Mitul Shah, Caroline Baynes, Don Conroy, Bridget Curzon, Patricia Harrington, Sue Irvine, Clare Jordan, Craig Luccarini, Rebecca Mayes, Hannah Munday, Barbara Perkins, Radka Platte, Anabel Simpson, Anne Stafford, and Judy West. We thank Nick Martin and Grant Montgomery for access to the QIMR Australian control series, and Dale Nyholt, Anjali Henderson, Margie Wright, Lisa Bowdler, Sara Smith, Megan Campbell, and Scott Gordon for QIMR control sample collection, data processing, and management.

Data on glycemic traits have been contributed by MAGIC investigators and have been downloaded from www.magicinvestigators.org.
