# Supplementary material for: Evidence of a Causal Association Between Insulinemia and Endometrial Cancer: A Mendelian Randomization Analysis
Source: J Natl Cancer Inst. 2015 Jul 1;107(9):djv178. doi: 10.1093/jnci/djv178 (PMC4572886; doi:10.1093/jnci/djv178)
Supplement: Supplementary Data [file supp_107_9_djv178__index.html]

Supplementary Data 

# Evidence of a Causal Association Between Insulinemia and Endometrial Cancer: A Mendelian Randomization Analysis

## Supplementary Data

Data files

- Supplementary Data - Supplementary Data
- Supplementary Data - Supplementary Data
