# Supplementary material for: Evidence of a Causal Association Between Insulinemia and Endometrial Cancer: A Mendelian Randomization Analysis
Source: J Natl Cancer Inst. 2015 Jul 1;107(9):djv178. doi: 10.1093/jnci/djv178 (PMC4572886; doi:10.1093/jnci/djv178)
Supplement: Supplementary Data [file supp_djv178_15_0015R1_Scott_supp_mat_AB_RS_KN_changes_accepted.docx]

**Supplementary materials**

**Endometrial cancer study case and control ascertainment**

**Cases**

**ANECS**

The Australian National Endometrial Cancer Study (ANECS) is an Australian population-based case-control family study of cancer of the uterine corpus^1^. Women aged 18-79, newly diagnosed with histologically confirmed primary cancer of the endometrium between July 2005 and December 2007 were identified through major hospitals nationally, and also from state-based cancer registries. Excluding women who could not be contacted (mostly due to death, illness or failure to contact), case participation rate was 63%. Participants completed a detailed questionnaire providing clinical and epidemiological information, including ethnicity of all four grandparents. Information on tumor pathology characteristics was abstracted in standardized format from clinical pathology reports for all patients.

**SEARCH**

The Studies of Epidemiology and Risk factors in Cancer Heredity (SEARCH) is an ongoing population-based study with cases ascertained through the Eastern Cancer Registration and Information Centre (http://www.ecric.org.uk). All women diagnosed with endometrial cancer between the ages of 18-69 years (average age of diagnosis 58 years) from August 2001 to September 2007 were eligible for inclusion. Approximately 54% of eligible patients have enrolled in the study. Women taking part in the study were asked to provide a 20ml blood sample for DNA analysis, and to complete a comprehensive epidemiological questionnaire. Controls were also drawn from SEARCH (http://ccge.medschl.cam.ac.uk/search/), but had no prior history of cancer at the time of recruitment. They were female, also between the ages of 18-69 at the time of recruitment and matched to cases in geographical profile. Approximately 35% of eligible controls enrolled in the study. All participants reported Caucasian ethnicity. Information on tumor pathology characteristics was provided by the Eastern Cancer Registration and Information Centre and was derived from clinical pathology reports for all patients.

Genome-wide genotyping of the ANECS and SEARCH cases was performed using an Illumina Infinium 610K array and called using the Illuminus algorithm. Genotypes were available for 1317 cases with endometrial cancer. Samples were excluded as follows: probable Turner’s syndrome or male sex based on genotypes for markers on the X and Y chromosomes (n=4); call rate <95% (n=15); heterozygosity outside 5 standard deviations from the mean (n=7); probable sibling pairs identified as close relatives by identity-by-state probabilities >0.85 (n=3); >15% non-European ancestry estimated from identity-by-state scores (n=1), leaving a total of 1287 cases (606 from ANECS and 681 from SEARCH). The duplicate concordance was 99.998%.

**Controls**

**QIMR**

The QIMR Berghofer Medical Research Institute control sample^2^ is a subsection of individuals recruited as part of the Brisbane Adolescent Twin Study^3,4^. Twins were recruited from schools in Brisbane, Australia and surrounding areas of southeast Queensland and were examined close to their 12th birthday. Blood was obtained from all twins and most parents. Parents were asked the ancestry of all eight great-grandparents of the twins. More than 95% of great-grandparents were identified as being of northern European ancestry, mainly from Britain and Ireland. This analysis used genotype data from parents and siblings only, extracted from an existing Illumina 610K BeadChip genome-wide association scan^2^ and recalled using the Illuminus algorithm. After standard QC steps (as for the case data) 1,846 QIMR Berghofer controls were included in the analysis.

**HCS**

The Hunter Community Study (HCS) is a population-based cohort study consisting of men and women aged 55-85 years of age in Newcastle, New South Wales, Australia^5^. Participants were randomly selected from the NSW State electoral roll (listing on the electoral roll is compulsory in Australia) and contacted between December 2004 and December 2007. Non-English speaking persons and those living in a residential aged-care facility were ineligible for participation in the study. Participants were asked to complete five self-report questionnaires as well as attend the HCS data collection centre so clinical measures could be obtained. In total, 44.5% of eligible controls agreed to participate in this study. Genotype data for this study were extracted from an existing Illumina 610K BeadChip genome-wide association study scan and recalled using the Illuminus algorithm. After standard QC steps (as for the case data) 1,237 HCS controls were included in the analysis.

**WTCCC**

Controls utilized for the SEARCH study were genotyped as part of the Wellcome Trust Case Control Consortium (WTCCC2)^6^. These controls are drawn from two sources: 2,922 controls from the 1958 Birth Cohort (1958BC), a population-based study in the United Kingdom of individuals born in 1 week in 1958^7^; and 2,737 controls identified through the UK National Blood Service (NBS)^6^ (13). The analyses presented here are based on 2,694 1958BC and 2,496 NBS controls for which valid genotype data were available at the time of analysis.

**Supplementary Tables and Figures**

**Supplementary Table 1: Individual variant risk factor weights and endometrial cancer associations**

rs5219 was not available in summary statistics for 30-minute insulin ([www.magicinvestigators.org](http://www.magicinvestigators.org)), so the weight was taken from an available proxy in high linkage disequilibrium (rs5215; r2=0.94) (please see attached excel file)

**Supplementary Figure 1**. Association of each single nucleotide polymorphism with **A**) fasting insulin (FI), **B**) early insulin secretion (IS), and **C**) body mass index (BMI) with endometrial cancer. Effect estimates and 95% confidence intervals are shown.

**Supplementary Figure 2.** Association of a genetic score of fasting glucose-associated single nucleotide polymorphisms with fasting glucose and a range of potential confounders in the Fenland study^8^. 30 minute insulin was only available in the Ely study^9^, so the sample size is smaller. Given 55 tests (α=9.1x10^-4^), the score was only associated with fasting glucose. Associations with quantitative traits were tested by linear regression and with binary traits by logistic regression. All statistical tests were two-sided.

**Supplementary Figure 3.** Association of a genetic score of type 2 diabetes-associated single nucleotide polymorphisms with glycemic traits and a range of potential confounders in the Fenland study^8^. 30 minute insulin was only available in the Ely study^9^, so the sample size is smaller. Given 55 tests (α=9.1x10^-4^), the score was only associated with fasting glucose. The score has previously been demonstrated to be strongly associated with type 2 diabetes^11^. Associations with quantitative traits were tested by linear regression and with binary traits by logistic regression. All statistical tests were two-sided.

**Supplementary references**

1. Spurdle A, Webb P. Re: Excess of early onset multiple myeloma in endometrial cancer probands and their relatives suggests common susceptibility. *Gynecol Oncol*. 2008;109(1):153; author reply 154.

2. Painter JN, Anderson CA, Nyholt DR, et al. Genome-wide association study identifies a locus at 7p15.2 associated with endometriosis. *Nat Genet*. 2011;43(1):51–54.

3. McGregor B, Pfitzner J, Zhu G, et al. Genetic and environmental contributions to size, color, shape, and other characteristics of melanocytic naevi in a sample of adolescent twins. *Genet Epidemiol*. 1999;16(December 1997):40–53.

4. Zhu G, Duffy DL, Eldridge A, et al. A major quantitative-trait locus for mole density is linked to the familial melanoma gene CDKN2A: a maximum-likelihood combined linkage and association analysis in twins and their sibs. *Am J Hum Genet*. 1999;65:483–492.

5. McEvoy M, Smith W, D’Este C, et al. Cohort profile: The hunter community study. *Int J Epidemiol*. 2010;39(January):1452–1463. doi:10.1093/ije/dyp343.

6. Wellcome T, Case T, Consortium C. Genome-wide association study of 14,000 cases of seven common diseases and 3,000 shared controls. *Nature*. 2007;447(7145):661–78.

7. Power C, Elliott J. Cohort profile: 1958 British birth cohort (National Child Development Study). *Int J Epidemiol*. 2006;35(September 2005):34–41.

8. Rolfe EDL, Loos RJF, Druet C, et al. Association between birth weight and visceral fat in adults. *Am J Clin Nutr*. 2010;92(2):347–52.

9. Forouhi NG, Luan J, Hennings S, Wareham NJ. Incidence of Type 2 diabetes in England and its association with baseline impaired fasting glucose: the Ely study 1990-2000. *Diabet Med*. 2007;24(2):200–7.

10. Perry JRB, Day F, Elks CE, et al. Parent-of-origin-specific allelic associations among 106 genomic loci for age at menarche. *Nature*. 2014.

11. Langenberg C, Sharp SJ, Franks PW, et al. Gene-lifestyle interaction and type 2 diabetes: the EPIC interact case-cohort study. *PLoS Med*. 2014;11(5):e1001647.
